# Telehealth Implementation, Treatment Attendance, and Socioeconomic Disparities in Treatment Utilization in a Community Mental Health Setting During the COVID-19 Pandemic: A Retrospective Analysis of Electronic Health Record Data

**DOI:** 10.1089/tmr.2022.0005

**Published:** 2023-05-04

**Authors:** Jonathan Kris

**Affiliations:** Department of Psychology, University of Detroit Mercy, Detroit, Michigan, USA.

**Keywords:** telehealth, mental health, treatment attendance, socioeconomic status, COVID-19

## Abstract

**Background:**

Previous studies have found that the widespread implementation of telehealth for outpatient mental health treatment during the COVID-19 pandemic has been associated with reduced no-show rates and increases in total number of appointments. However, it is unclear to what degree this is due to increased accessibility provided by telehealth, rather than to increased consumer demand for services fueled by the pandemic-related exacerbation of mental health needs. To shed light on this question, the present analysis examined changes in attendance rates for outpatient, home-, and school-based programs at a community mental health center in southeastern Michigan. Disparities in treatment utilization associated with socioeconomic status were also examined.

**Methods:**

Two-proportion z-tests were conducted to examine changes in attendance rates, and Pearson correlations were calculated using the median income level and attendance rate by zip code to examine disparities in utilization associated with socioeconomic status.

**Results:**

The proportion of appointments kept after telehealth implementation was statistically significantly higher for all outpatient programs, but not for any home-based programs. Specifically, absolute increases in the proportion of appointments kept ranged from 0.05 to 0.18 for outpatient programs, representing relative increases of 9.2% to 30.2%. Furthermore, before telehealth implementation, there was a strong positive correlation between income and attendance rate for all outpatient programs (ranging from *r* = 0.50 to 0.56). After telehealth implementation, there were no longer any significant correlations.

**Discussion:**

Results highlight the utility of telehealth in increasing treatment attendance and mitigating disparities in treatment utilization associated with socioeconomic status. These findings are highly relevant to ongoing discussions surrounding the long-term fate of evolving insurance and regulatory guidelines pertaining to telehealth.

## Introduction

Telehealth, defined by the Health Resources and Services Administration as “the use of electronic information and telecommunication technologies to support long-distance clinical health care, patient and professional health-related education, health administration and public health,”^1^ saw rapid and widespread adoption as health care providers adapted their services to the unfolding circumstances surrounding the COVID-19 pandemic—particularly in March 2020, after the WHO's declaration of the outbreak as a global pandemic.

Mental health services, in particular, saw high rates of telehealth adoption.^2^ An American Psychological Association (APA) survey from June 2020 found that 92% of clinicians were providing services using telehealth, with 76% of clinicians using telehealth alone;^3^ compared with only 20% utilizing telehealth before the pandemic, as reported in a previous survey.^4^

Among studies examining the process and impact of this rapid telehealth implementation for mental health treatment, some of the most consistently reported outcomes have been reduced no-show rates and increases in total number of appointments.^3,5–8^ Existing studies have primarily focused on outpatient programs, and have typically compared 1–6 month periods before, and after, telehealth implementation.

Chen et al.,^5^ for example, using data from the Massachusetts General Hospital outpatient psychiatry division, reported a 20% decrease in no-shows from January and February 2020 to April and May 2020. Mishkind et al.,^6^ using data from two outpatient mental health clinics at the University of Colorado Anschutz Medical Campus, reported a 5.1% decrease in no-shows (11.9% in January and February 2020, to 6.8% in April–September 2020), as well as a 26.2% increase in overall completed appointments.

More broadly, an APA survey from November 2020 found that 44% of clinicians reported fewer no-shows or cancellations since adopting telehealth (with 42% reporting no change).^3^ One aim of the present analysis was to determine whether these findings would be replicated across a longer period of time (3 years before to 1 year after), by examining attendance data from a community mental health center in southeastern Michigan. Furthermore, given that little to no data on home- or school-based treatment programs has been reported yet in this respect, the present analysis also examined attendance data from home- and school-based mental health programs within the agency.

In addition to providing initial data on the effects of telehealth implementation for these programs, examining home- and school-based programs may provide some insight into an existing limitation in making inferences from the aforementioned findings: Specifically, in interpreting the observed increases in attendance, authors have generally cited the increased accessibility provided by telehealth as the primary factor driving these changes, for example, Chen et al.: “early quality improvement data from our department suggest that no-show rates have decreased by 20% … likely due to decreased logistical barriers to access.”^5^

Indeed, geographical and transportation-related barriers are well established as obstacles in accessing health care services for many people, particularly from rural and lower socioeconomic status communities;^9–11^ and the mitigation of these barriers is one of the central benefits of telehealth emphasized in its existing body of literature.^12–15^ However, it is also clear that the pandemic has resulted in a dramatic spike in the prevalence and severity of mental health conditions, as well as in the demand for treatment for them.^3,16–20^

Therefore, it is not yet completely clear how much of the increase in service utilization is due to increased accessibility provided by telehealth, rather than to an increase in consumer demand for services fueled by the general pandemic-related exacerbation of mental health needs. To consider in brief: Between 29% and 42.6% of adults reported symptoms of anxiety disorder and/or depressive disorder during the months of May 2020 to July 2021, compared with only 10.8% of adults over 2019, according to survey data from the U.S. Census Bureau and National Center for Health Statistics^16,17^—trends that were similarly observed in a recent systematic review.^18^

Regarding demand for services, a survey conducted by the National Council for Mental Wellbeing found that 52% of behavioral health organizations reported an increase in demand for services through August 2020, and that this number had increased to 67% by February 2021.^19^ An APA survey from November 2020 found that 37% of clinicians reported an increase in referrals,^3^ and the Substance Abuse and Mental Health Services Administration saw an 890% increase in the number of people calling its Disaster Distress Helpline in the initial months after March 2020.^20^

Examining outpatient, home-, and school-based programs may offer some insight into this question, in that if increases in service utilization are similarly present in programs where barriers associated with traveling to the provider have already been effectively eliminated (e.g., home-based programs), this may provide some initial evidence for the relevance of factors other than increased accessibility in contributing to increased attendance.

Conversely, to the extent that increases in service utilization are present uniquely in outpatient programs, this could provide evidence for the aforementioned hypothesis that increased accessibility is the primary factor driving the changes in mental health service utilization (in that it would confirm an observation reasonably expected given said hypothesis). Finally, given the known associations between lower socioeconomic status and decreased utilization of mental health services, and that telehealth is hypothesized to mitigate one of the central barriers to access for this group, data were analyzed to determine whether any disparities in utilization associated with socioeconomic status (if present) had been decreased.

## Background

At the community mental health center in southeastern Michigan from which data were analyzed, psychotherapy, psychiatry, case management, and other mental health treatment services are provided for children, adolescents, and adults on an outpatient basis as well as through home-based treatment modalities such as general home-based therapy (ages 7–17), Infant Mental Health/Early Childhood (IMH/EC) services (ages 0–6), and Wraparound (treatment and personal support services for individuals ages 0–21). Patients present with a wide variety of psychiatric disturbances and duration of treatment is highly variable, particularly in outpatient programs where patients may drop out shortly after intake or remain in treatment for one or more years.

For all home-based services, clinicians travel to the family's home or specified location within the community. School-based services are also provided, in which clinicians meet with students attending the school at which the clinician is located. The majority of these services are based out of a single site. However, this site serves all of Wayne county and has a diverse clientele. For instance, for the adult outpatient program, there were 49 zip codes from which at least 100 appointments were scheduled both before and after the pandemic, with the median income for these zip codes ranging from $17,331 to $101,944.

## Methods

A retrospective analysis of electronic health record (EHR) data was conducted to examine the proportion of appointments kept before and after telehealth implementation. All appointments were virtualized (i.e., telehealth was implemented) beginning March 20, 2020. The year after telehealth implementation (March 20, 2020–March 19, 2021) was compared with the 3 years directly prior (March 20, 2017—March 19, 2020). This analysis was approved by the agency's Institutional Review Board (IRB).

Eight programs were analyzed separately: child outpatient therapy, child psychiatry, adult outpatient therapy and case management, adult psychiatry, Wraparound, IMH/EC, home-based therapy, and school-based therapy. Appointments were grouped into these categories using the listed billing code/designated purpose of appointment (using the conventions of each program), the assigned staff's primary role (Table 1). Appointments with purposes/billing codes other than those listed, or where staff role was unclear, were excluded.

**Table 1.** Grouping of Electronic Health Record Appointments by Program

Program	Any appointments with staff from that program marked as
Child outpatient	“90837,” “90834,” “90832,” “9083X,” “90847,” “90846,” “9084X,” “T1017,” “Case management/therapy,” “Intake,” “Annual Paperwork,” or “First Service”
Child psychiatry	“Psychiatric Evaluation,” “Medication Review,” or “Emergency Medication Review”
Adult outpatient	“90837,” “90834,” “90832,” “9083X,” “90847,” “90846,” “9084X,” “T1017,” “Case management/therapy,” “Intake,” “Annual Paperwork,” or “First Service”
Adult psychiatry	“Psychiatric Evaluation,” “Medication Review,” or “Emergency Medication Review”
Home based	“H0036,” “Case management/therapy,” “Intake,” “Annual Paperwork,” or “First Service”
Infant mental health/early childhood	“H0036,” “Case management/therapy,” “Intake,” “Annual Paperwork,” or “First Service”
Wraparound	“H2021,” “H2015,” or “First Service”
School based	“90837,” “90834,” “90832,” “90847,” “90846,” “H0031,” “H0032,” “Case management/therapy,” “Intake,” “Annual Paperwork,” or “First Service”

Rather than comparing rates of no-shows, the proportion of appointments “kept” (all appointments marked “kept”) and “not kept” (all appointments not marked “kept”) was examined, as this seemed to constitute the most reliable marker of appointment status, given some discrepancies in how EHR appointments are marked between staff/programs.

To examine attendance rates, a two-proportion z-test was conducted for each program to compare the proportion of appointments kept before and after telehealth implementation. In keeping with statistical reporting recommendations for large electronic health record data sets,^21^ a measure of effect size (Cohen's H) was calculated as well.

To examine the relationship between socioeconomic status and attendance rate, for each program, a Pearson product-moment correlation coefficient was calculated using the median income level and attendance rate for each zip code from which at least 100 appointments were scheduled both before and after telehealth implementation. Median income for each zip code was obtained using data from the U.S. Census Bureau. Owing to the limited number of zip codes for some programs, data were pooled where feasible; specifically, child outpatient therapy and child psychiatry were combined, and all home-based programs were combined. School-based data were not included due to having a prohibitively limited number of zip codes from which at least 100 appointments were scheduled both before and after telehealth implementation (5).

To examine the significance of potential changes in these correlations after implementation of telehealth, correlations were transformed into z-scores using Fisher's r-to-z transformation, and a z-score based on the difference between these values (and the variance of the difference between them) was calculated to assess the degree and statistical significance of any changes (cf. Esterberg et al.^22^). All statistical procedures were performed using R version 4.0.3.^23^

## Results

Attendance rates after telehealth implementation were statistically significantly higher than attendance rates before telehealth implementation for all outpatient programs, with increases ranging from 9.2% to 30.2% (Table 2). However, for all home-based programs, there were no statistically significant differences in attendance rates (changes ranged from 0.7% to −1.2%). School-based therapy saw a statistically significant drop in attendance (−5.5%).

**Table 2.** Two-Proportion z-Tests for Appointments Kept Before and After Telehealth Implementation

	*N*	Pre-telehealth	Post-telehealth	Absolute change (as proportion)	Relative change (as percentage)	Z-score	Effect size (Cohen's H)
Child outpatient	27,990	0.56	0.65	0.09^***^	16.3	13.63	0.19
Child psychiatry	8298	0.57	0.75	0.18^***^	30.2	13.26	0.37
Adult outpatient	133,037	0.56	0.69	0.13^***^	22.7	42.94	0.27
Adult psychiatry	100,634	0.59	0.64	0.05^***^	9.2	13.33	0.11
Home based	12,135	0.79	0.79	0.00	0.7	0.72	0.01
Infant mental health/early childhood	3156	0.72	0.72	0.00	0.9	0.35	0.01
Wraparound	3325	0.85	0.84	−0.01	−1.2	−0.82	0.03
School based	17,497	0.81	0.77	−0.04^***^	−5.5	−6.91	0.11

*α* = 0.05.

^***^ *p* < *0*.001, two-tailed.

Using Cohen's H as a measure of effect size, the changes in child outpatient and adult outpatient would be considered approximately small effects, with the change in adult psychiatry being a small-to-medium effect, using the conventions proposed by Cohen (although the utility of these conventions is context dependent, with exact values ultimately being preferred for assessing clinical significance).^24^

Before telehealth implementation, there was a strong positive correlation between socioeconomic status and attendance rate for child outpatient therapy/psychiatry (*r*[18] = 0.49, *p* = 0.028), adult outpatient therapy and case management (*r*[32] = 0.57, *p* < 0.001), and adult psychiatry (*r*[47] = 0.51, *p* < 0.001), and a strong negative correlation for home-based programs (*r*[13] = −0.54, *p* = 0.039) (Table 2). After telehealth implementation, there were no longer any significant correlations between socioeconomic status and attendance rate for any programs (Table 3).

**Table 3.** Pearson Product-Moment Correlation Coefficients for Median Income and Attendance Rate by Zip Code, with z-Scores Based on the Differences Between Correlations Transformed Using Fisher's r-to-z Transformation

	*N*	Pre-telehealth Pearson's r	Post-telehealth Pearson's r	Z-score
Adult outpatient	49	0.51^***^	−0.12	3.247^***^
Adult psychiatry	34	0.57^***^	0.01	2.514^**^
Child (outpatient and psychiatry)	20	0.49^*^	0.27	0.742
Home based (all)	15	−0.54^*^	−0.10	−1.214

Pearson's *r*, two-tailed; z-scores, one-tailed. *α* = 0.05.

^*^ *p* < 0.05, ^**^p < 0.01, ^***^p < 0.001.

The change in correlation between socioeconomic status and attendance rate (from before to after telehealth implementation) was found to be statistically significant for adult psychiatry [*z* = 2.51, *p* < 0.01] and adult outpatient therapy and case management [*z* = 3.25, *p* < 0.001]. A nonsignificant change in the predicted direction was found for child outpatient therapy and psychiatry [*z* = 0.74, *p* = 0.49]. The change in home-based programs was also not significant, but trended in the opposite direction [*z* = −1.21, *p* = 0.11].

## Discussion and Limitations

Results of the present analyses are broadly consistent with the findings and corresponding hypotheses of previous authors in that, (1) significant increases in attendance rates were similarly observed across multiple outpatient programs after the implementation of telehealth, and (2) as would be expected if increased accessibility were a central factor driving these changes, the increases in attendance rates were seen only in outpatient programs (where accessibility is known to be a central barrier), with there being no significant changes among home- or school-based programs (which are already largely structured so as to eliminate these barriers).

In addition, the fact that all home- and school-based programs had higher rates of attendance than outpatient programs before telehealth implementation suggests that the measures taken by home- and school-based programs to increase treatment utilization may indeed have been substantially effective. The significant drop in attendance for school-based services is likely due to school closures resulting in disruptions to school-based therapists' primary vehicle for engaging youth and their families in services (although services were still available by phone or video during these times).

Another significant finding of the present analysis is the fact that telehealth implementation in outpatient programs was associated not only with increased attendance generally, but also with a reduction in socioeconomic status-related disparities in utilization. This suggests that telehealth may have been particularly useful for increasing utilization among individuals from lower socioeconomic status backgrounds.

Although this trend was found for all outpatient programs, one limitation of this part of the analysis was that the correlation analysis for home-based programs was significantly underpowered due to the limited number of zip codes available. Thus, it is difficult to draw conclusions about the extent to which this trend may have actually been present among home-based programs as well. However, the fact that there were no significant changes in attendance among any of the home-based programs suggests that this possibility may be unlikely.

Although these results examine the extent to which initially observed trends related to telehealth implementation have persisted over the year after, circumstances surrounding the COVID-19 pandemic are continually evolving, and future study should examine the extent to which findings from the first year have persisted throughout the second, and continue to after.

These results provide additional evidence for the utility of telehealth in increasing treatment attendance and mitigating disparities in treatment utilization associated with socioeconomic status—findings that will be highly relevant to ongoing discussions surrounding the long-term fate of evolving insurance and regulatory guidelines pertaining to telehealth moving forward.

## Abbreviations Used

APA: American Psychological Association
HER: electronic health record
IMH/EC: Infant Mental Health/Early Childhood
